# Branched Linkers for Site-Specific Fluorescent Labeling of Antibodies

**DOI:** 10.3390/molecules28010425

**Published:** 2023-01-03

**Authors:** Ksenia A. Sapozhnikova, Evgeny L. Gulyak, Vsevolod A. Misyurin, Maria A. Simonova, Ekaterina V. Ryabukhina, Anastasiya V. Alexeeva, Nataliya A. Tikhonova, Natalia A. Lyzhko, Galina P. Popova, Andrey V. Misyurin, Alexey V. Ustinov, Vladimir A. Korshun, Vera A. Alferova, Dmitry Yu. Ryazantsev, Vladimir A. Brylev

**Affiliations:** 1 Shemyakin-Ovchinnikov Institute of Bioorganic Chemistry, Miklukho-Maklaya 16/10, 117997 Moscow, Russia; 2 N.N. Blokhin National Medical Cancer Research Center, Ministry of Health of Russia, Kashirskoye sh. 24, 115478 Moscow, Russia; 3 GeneTechnology LLC, Profsoyuznaya 104, 117485 Moscow, Russia; 4 Lumiprobe RUS Ltd., Kotsyubinskogo 4, 121351 Moscow, Russia

**Keywords:** fluorescent antibody, branched linkers, FRET, degree of labeling, periodate oxidation, immunoglobulin G, flow cytometry, PRAME, antibody conjugate

## Abstract

Fluorescent antibodies have proved to be an invaluable tool for molecular biology and diagnostics. They are routinely produced by modification of lysine residues, which leads to high heterogeneity. As such, their affinity may be compromised if the antigen-binding site is affected, the probability of which increases along with the degree of labeling. In this work, we propose a methodology for the synthesis of site-specific antibody-dye conjugates with a high degree of labeling. To this end, we synthesized two oxyamine-based branched triazide linkers and coupled them with a periodate-oxidized anti-PRAME antibody 6H8; two oxyamine-based linear monoazide linkers of similar structure were used as controls. The azide-labeled antibodies were subsequently conjugated with fluorescent dyes via SPAAC, a copper-free click reaction. Compared to their counterparts made with linear linkers, the branched conjugates possessed a higher degree of labeling. The utility of the methodology was demonstrated in the detection of the PRAME protein on the surface of the cell by flow cytometry.

## 1. Introduction

Fluorescently labeled antibodies are a powerful tool for various analytical and diagnostic approaches, such as flow cytometry and immunocytochemistry [1,2]. Most often, modified antibody synthesis is based on conjugation via maleimide-thiol or active ester-amino group chemistry. Both approaches are not free of flaws: acylation of lysine residues may compromise the affinity if the antigen-binding site is modified, while the reduction of disulfide bonds followed by conjugate addition can impact the tertiary structure of the antibody [3,4]. In addition, these conjugation methods yield highly heterogeneous mixtures of products. For these reasons, site-specific modification of antibodies is regarded as preferable [5,6].

Most existing methodologies for site-specific antibody modification are rather intricate, relying on the introduction of non-natural amino acids [7], enzymatic remodeling of glycans [8], or proximity-induced chemistry [9]. In contrast, glycan modification via periodate oxidation followed by reacting the resulting aldehyde groups with nucleophiles constitutes a much simpler approach [10,11,12]. This methodology is at its best when coupled with oxime ligation, which yields stable conjugates without the need for C=N double bond reduction [13].

**Figure 1:**
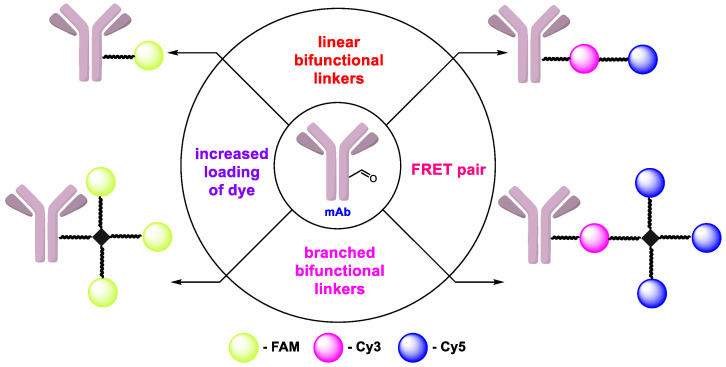
Fluorescent antibodies prepared in this study using linear and branched linkers.

Here, we describe a labeling technique based on periodate oxidation–oxime ligation for the purpose of introducing azido groups into an antibody, followed by strain-promoted azide-alkyne cycloaddition (SPAAC) click modification with fluorescent dyes. This approach affords site-specifically modified fluorescent antibodies with controllable stoichiometry. In particular, the use of bifunctional reactive dye derivatives and branching reagents yielded conjugates with a higher degree of labeling (DOL). We aimed at probing the utility of the branching reagents by directly comparing them to linear bifunctional reagents of similar structure in terms of the degree of labeling of the antibody and its fluorescent properties (Figure 1). Since the most common laser channels are compatible with fluorescein (FAM), Cy3, and Cy5 cyanines, we chose these three dyes for model conjugates.

## 2. Results and Discussion

### 2.1. Synthesis of Azide Linkers

**Scheme 1:**
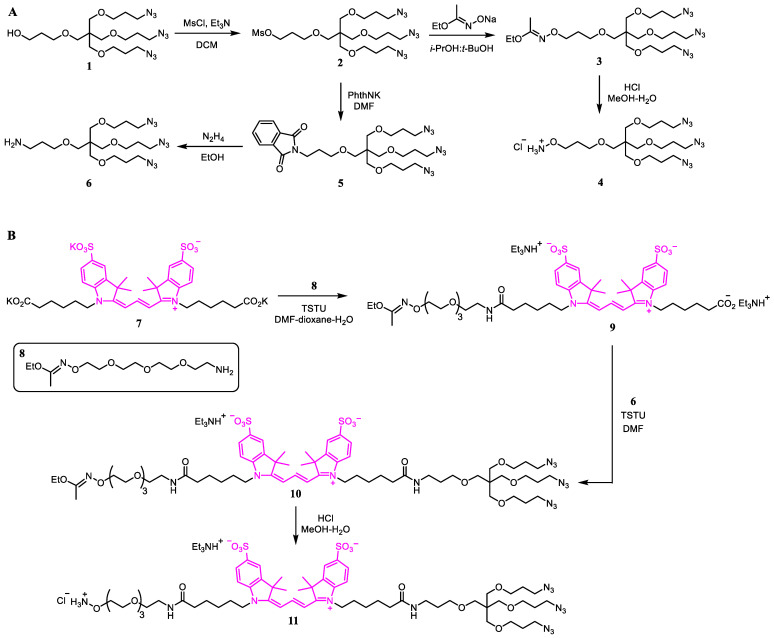

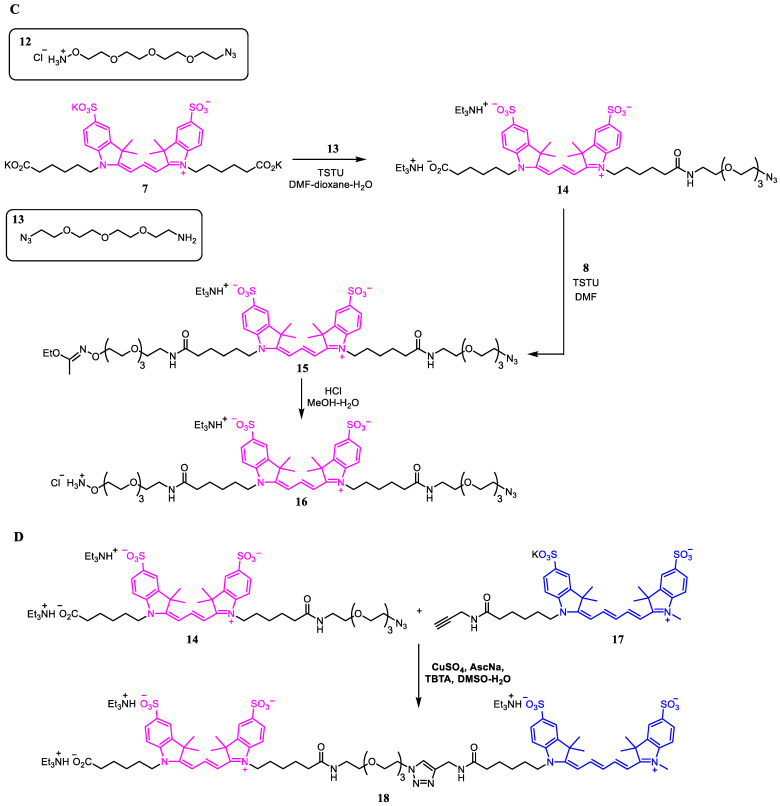
(**A**) Synthesis of the branched triazide reagent **4** and the building block **6**. (**B**) Synthesis of the fluorescent branched linker **11**. (**C**) Synthesis of the linear reagent **16** and the structure of the known linker **12**. (**D**) Synthesis of the sCy3-sCy5 conjugate **18**.

We used the previously developed pentaerythritol-based triazide **1** [14] as the starting material for creating new bifunctional branched cores (Scheme 1). The synthesis commenced with making the key compound **2**. This mesylate was prepared from alcohol **1** using mesyl chloride and an appropriate base. Although in the present work, it was purified by column chromatography for characterization purposes, we found that its use in the next synthetic step does not require purification. Next, we obtained ethoxyethylidene-protected oxyamine **3** from the mesylate and sodium salt of ethyl-N-hydroxyacetimidate according to the method described earlier (Scheme 1A). This protected oxyamine core can be used as a branching reagent for antibody conjugates. In our case, it was employed to increase the loading of fluorescent dyes (Figure 1). The protecting group can be removed in situ, and ligation with carbonyl groups can instantly occur as we described earlier [15] by incubation with previously oxidized antibodies at pH 3–4.5. It is a convenient method for dyes unstable in the presence of a free oxyamino group, e.g., fluorescein. It should be noted, however, that prolonged incubation of antibodies at pH values of 3 or lower can lead to reduced affinity, even though the oxyamine deprotection occurs efficiently under these conditions. To this end, we found that the optimal pH value for in situ deprotection/oxime ligation is 4.0. At this pH, oxyamine deblocking and subsequent ligation occurs without significant damage to the integrity of the antibody. With a sufficient excess of the dye (~100 equiv.) and an incubation time of about 1 h, it is possible to achieve an antibody labeling degree of 2–4.

On the other hand, many dyes can tolerate the presence of a protonated/free aminooxy group for some period of time. Should such dyes be used for labeling, incubation of the antibody at a more basic pH is possible. A suitable buffer for oxime ligation is the acetate buffer with a pH of 5.0. At this level of acidity, the periodate oxidation is at its best, and the side reaction of the formation of Schiff bases of the carbonyl groups with lysine residues is suppressed [16]. In contrast to the formation of Schiff bases, which proceeds better in a slightly alkaline environment, oxime ligation reactions are best performed under acidic conditions. Considering that, we also made the deprotected branched derivative **4** from **3** by incubation in methanolic HCl (Scheme 1A).

From mesylate **2** we obtained another branched bifunctional linker **6** with an amino group instead of the oxyamino group. For this purpose, the mesylate was converted to phthalimide **5**, followed by deprotection with hydrazine (Scheme 1A). The bifunctional linker **6** itself may be of interest for different applications. In our case, it was condensed with the sulfo-Cy3 derivative **9** to yield the bifunctional dye-containing linker **10** (Scheme 1B).

The conversion of **7** to **9** was performed using the water-tolerant coupling reagent TSTU in a DMF–1,4-dioxane–H_2_O triple solvent system. This combination of solvents was chosen to improve the solubility of the metal salt **7**. First, the monocondensation product **9** was obtained by desymmetrization (a mixture of mono and bis products was synthesized and separated by column chromatography). It was then reacted with reagent **6** to yield **10** (Scheme 1B). Acidic deprotection yielded the free oxyamine variant of the fluorescent linker **11** along with linkers **12** and **16**, the latter being obtained from **7** (Scheme 1C).

### 2.2. Synthesis of Antibody Conjugates

**Scheme 2:**
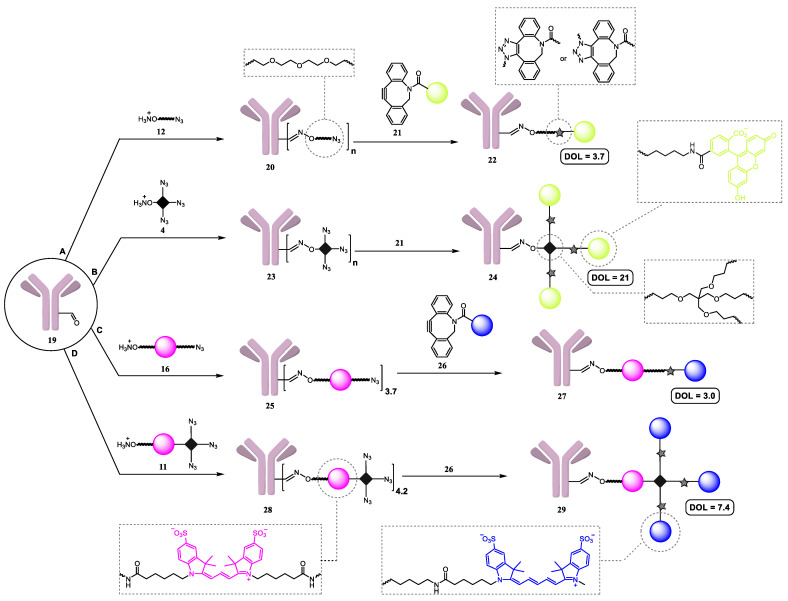
Synthesis of fluorescent antibody conjugates.

For the present work, we chose the anti-PRAME antibody 6H8 [17], which we had previously used in our studies [15,18]. The PRAME protein is expressed on the surface of melanoma and leukemia cells and is thus a promising target for diagnostic and therapeutic tools [19,20,21,22,23]. We set out to obtain antibody conjugates with linear and branched reagents (both dye-based and not) and then modify them with dyes using strain-promoted azide-alkyne cycloaddition (SPAAC) (Scheme 2). SPAAC was chosen, as it is less likely to compromise the antibody’s affinity compared to copper-catalyzed azide-alkyne cycloaddition (CuAAC) [24].

First, we obtained antibodies labeled with branched reagent **4** (Scheme 2A,B). For comparison, we also obtained an antibody labeled with linear linker **12**. For that, we oxidized the antibodies with sodium periodate in an acetate buffer in a weakly acidic medium. Beforehand, we studied the effect of periodate concentration and oxidation time on the affinity of antibodies. The conclusion was that the most optimal is the use of 20 mM sodium periodate for 30 min in acetate buffer with a pH value of 5. Upon oxidation, the antibody was desalted and incubated with linear reagent **12** and branched reagent **4** in a weakly acidic acetate buffer for 1 h.

The resultant conjugates were purified and reacted with the fluorescein dibenzocyclooctyne (DBCO) derivative **21**, which is suitable for effective click reaction with azides in biomolecules, to yield the linear and branched FAM conjugates **22** and **24**. In a similar way, we obtained conjugates **25** and **28** with fluorescent linkers **16** and **11**, respectively (Scheme 2C,D). They were then modified in a SPAAC click reaction with the sulfo-Cy5 DBCO dye **26**. Due to the hydrophobicity of the DBCO groups, the final conjugates exhibited instability and were prone to aggregation in PBS. In order to overcome this problem, we used 0.01% Tween 80 in PBS as a stabilizer for the linear conjugates (**22**, **27**) and 0.05% Tween 80 for the branched ones (**24**, **29**). In the presence of Tween 80, the conjugates were found to be stable for at least a week at +4 °C.

### 2.3. Degree of Labeling

**Figure 2:**
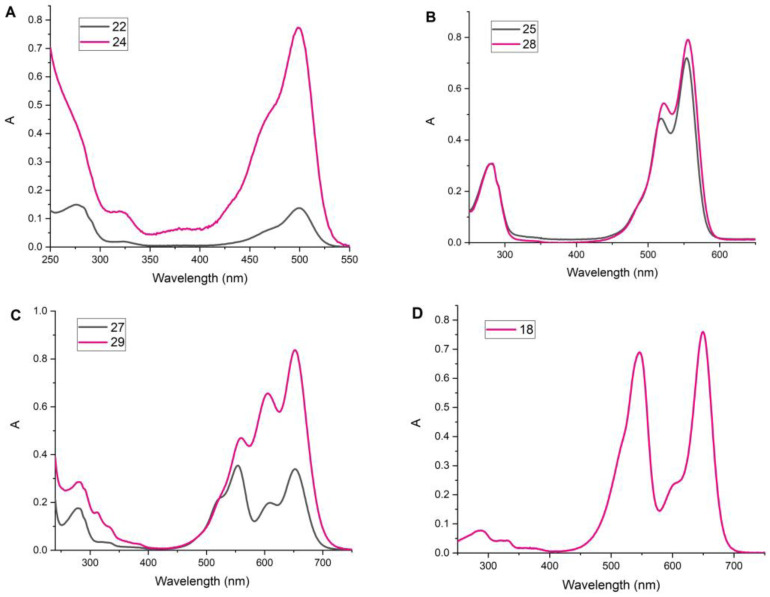
(**A**–**C**). UV–Vis spectra of the FAM- (**22** and **24**), sCy3- (**25** and **28**), and sCy3-sCy5 (**23** and **25**) derivatives of antibody 6H8 normalized by antibody concentration. (**D**) Absorbance spectrum of the sCy3-sCy5 conjugate **18**.

The stoichiometry of the dye conjugates was determined by UV–Vis spectrophotometry using the molar absorbance coefficients listed in Table 1. The degree of modification of the conjugates **20** and **23** could not be measured by this method, so it was assessed indirectly by measuring the DOL of their fluorescein derivatives **22** and **24**. Somewhat surprisingly, even though **20** and **23** were synthesized under virtually identical conditions, **23** appeared to have a substantially higher degree of modification, giving rise to conjugate **24** with a DOL of at least 21 (see Section 4 for details) as compared with its linear counterpart **22** (DOL = 3.7) (Figure 2A).

In contrast, conjugates **25** and **28** synthesized under similar conditions have a comparable degree of modification (Figure 2B) that can be measured directly, which suggests that they are better suited for the controlled introduction of azide moieties into antibodies. To obtain a reference for the 100% yield of the click reaction, we synthesized the low-molecular-weight sCy3-sCy5 conjugate **18** (Scheme 1D) and measured its sCy3/sCy5 absorbance ratio at their maxima (Figure 2D). Surprisingly, it turned out that the value (0.91) was much higher than expected ([ε_548_ sCy3+ε_548_ sCy5]/ε_646_ sCy5 = (1.62 × 10^5^ + 1.20 × 10^4^) M^−1^cm^−1^/(2.71 × 10^5^) M^−1^cm^−1^ = 0.64). This was used to evaluate the degree of modification of **25** with sCy5-DBCO, which proceeded with 82% yield, giving rise to conjugate **27** with a DOL of 3.0. Under analogous conditions, branched conjugate **28** yielded conjugate **29** that possessed a higher DOL of 7.4 as measured by sCy5 (Figure 2C).

### 2.4. Study of Fluorescent Properties

**Figure 3:**
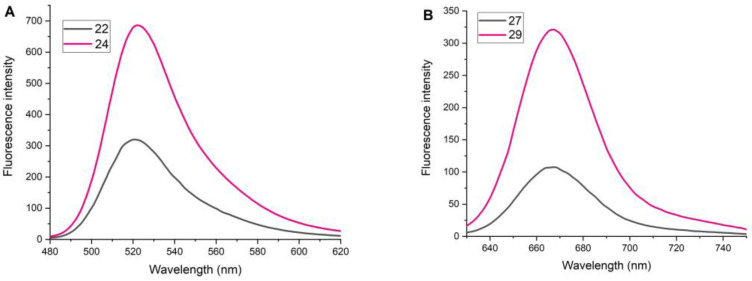

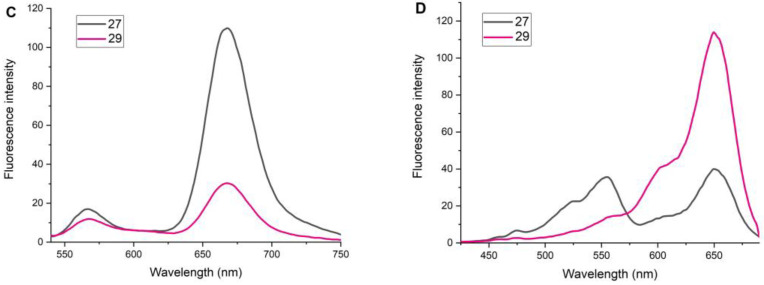
Fluorescence spectra of 6H8 conjugates normalized by 6H8 concentration. (**A**) Emission spectra of **22** and **24**, excitation wavelength: 460 nm. (**B**) Emission spectra of **27** and **29**, excitation wavelength: 610 nm. (**C**) Emission spectra of **27** and **29**, excitation wavelength: 520 nm. (**D**) Excitation spectra of **27** and **29**, emission wavelength: 710 nm.

In order to evaluate the fluorescent properties of the labeled antibodies, fluorescence emission spectra of the four final conjugates were recorded. While branched conjugate **24** demonstrated higher fluorescence intensity compared to **22** (Figure 3A), the approximately twofold increase was disproportionate to the more than fivefold difference in the DOL (21 vs. 3.7); the discrepancy can be ascribed to self-quenching of fluorescein due to fluorophores being in close proximity around the branched core. Contrarily, the difference in fluorescence intensity in conjugates **29** and **27** was completely proportional to the difference in the degree of labeling, indicating that no apparent self-quenching took place (Figure 3B). This might be in part due to a longer linker connecting dye fragments to the core in **29** compared to **24** (Scheme 2).

For additional proof of the structure of conjugates **27** and **29**, we studied the FRET effect in the sCy3-sCy5 pair (Figure 3C,D). Energy transfer was observed in both conjugates; curiously, it was found to be more efficient in **27** compared to **29** despite the lower number of sCy5 fragments per one sCy3.

### 2.5. Antibody Conjugate Affinity Determination

**Figure 4:**
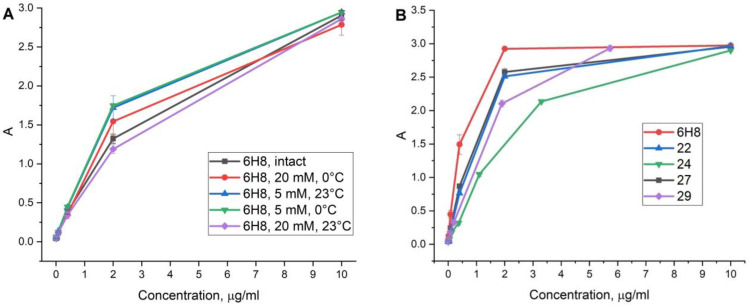
Determination of affinity by ELISA. (**A**) Characterization of the affinity of antibody 6H8 in different oxidation conditions (variable concentrations of NaIO_4_—5 and 20 mM, the variable temperature of reaction—0 °C and 23 °C, constant time—30 min; after completion of the reaction, oxidized antibody was treated with semicarbazide). (**B**) Characterization of the affinity of the linear and branched antibody conjugates.

We studied the effect of sodium periodate concentration and temperature on the affinity of the modified 6H8 antibody. For this purpose, 6H8 was oxidized at two different periodate concentrations, 5 mM and 20 mM, at room temperature and at 0 °C (Figure 4A). The buffer chosen was 20 mM acetate with pH 5.0 and 150 mM NaCl. In all cases, the reaction proceeded for 30 min. After which, excess periodate was quenched with a 20% solution of glycerol in water. The antibodies were purified by gel filtration and then incubated with semicarbazide for 60 min in an acetate buffer at pH 5.0. The modified antibodies were then analyzed by ELISA. It was found that in the case of antibody 6H8, the periodate concentration in the range of 5–20 mM had no significant effect on the affinity. Similarly, the temperature had no effect, and in all cases, the antibody retained its affinity. Based on the data obtained, we chose 20 mM sodium periodate concentration, room temperature, incubation for 30 min, and an acetate buffer with pH 5.0 to obtain the fluorescent conjugates. The affinity of the four final conjugates, also determined by ELISA, was shown to be largely uncompromised (Figure 4B).

### 2.6. Flow Cytometry

**Figure 5:**
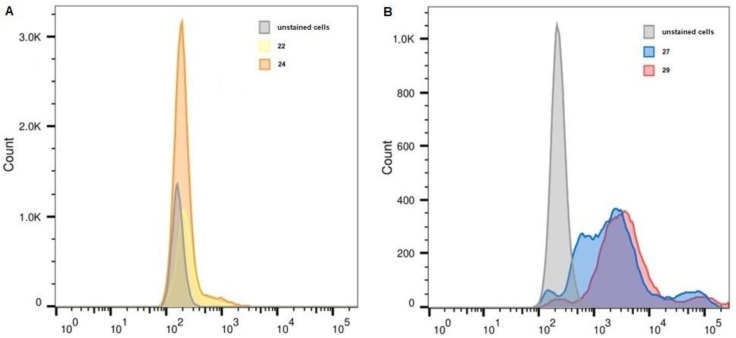
K-562 cells staining. (**A**) FITC channel (fluorescein). (**B**) APC channel (sCy5).

**Figure 6:**
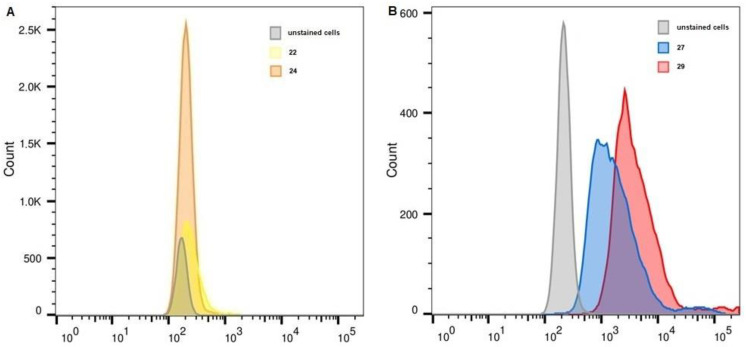
THP-1 cells staining. (**A**) FITC channel (fluorescein). (**B**) APC channel (sCy5).

**Figure 7:**
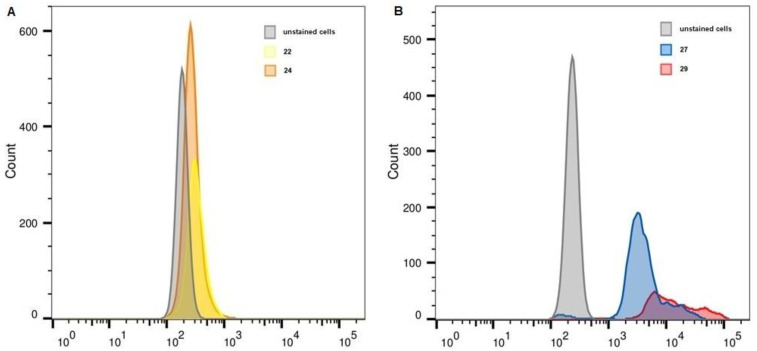
MelP cells staining. (**A**) FITC channel (fluorescein). (**B**) APC channel (sCy5).

**Figure 8:**
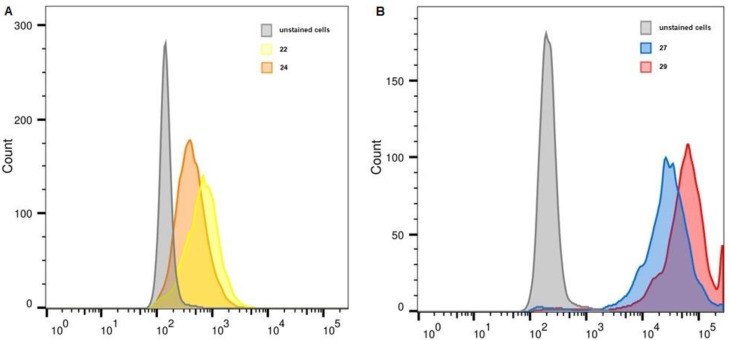
WI-38 PRAME+ cells staining. (**A**) FITC channel (fluorescein). (**B**) APC channel (sCy5).

The four fluorescent conjugates were used to detect PRAME-expressing cells (lines THP-1, K562, MelP, and WI-38 with PRAME overexpression [25]) in flow cytometry experiments. The median fluorescence intensity of the stained cells was determined, and the unstained cells were used as controls (Figure 5, Figure 6, Figure 7 and Figure 8). The data is summarized in Table 2.

For fluorescein-based conjugates **22** and **24**, fluorescence was registered in the FITC channel. For lines K-562, THP-1, and MelP, the difference between the stained cells and the control was marginal, and sufficient fluorescence was only observed for PRAME-overexpressing WI-38 (Figure 7A). The branched conjugate **24** was found to perform worse than its linear counterpart **22** (Table 2), which we believe is due to a combination of factors: (1) self-quenching of fluorescein (as described in Section 2.4) and (2) lower affinity of **24** compared to **22** (Figure 3B). It should also be noted that for high-DOL conjugates, the mean DOL of the cell-bound fraction can be lower than that of the free conjugate, as affinity is generally inversely correlated with the DOL [12]; should this be the case, the fluorescence of stained cells becomes lower than expected.

In the case of conjugates **27** and **29**, sufficient fluorescence was registered for all cell lines in the APC channel. Contrary to what was observed for fluorescein-labeled antibodies, the branched conjugate **29** consistently performed better than **27**, giving rise to cell fluorescence intensity that was 1.6 to 3 times higher (Table 2). In this case, the higher DOL was successfully translated into increased cell fluorescence thanks to the lack of self-quenching in **29** and the small difference in the affinity of **27** and **29**.

## 3. Materials and Methods

### 3.1. General Methods

Dimethylformamide (DMF) was purified by distillation with benzene/water followed by vacuum distillation over CaH_2_ and stored over 3 Å molecular sieves. Dichloromethane (DCM) and methanol (MeOH) were purified by distillation; dimethyl sulfoxide (DMSO) was purified by vacuum distillation and stored over 3 Å molecular sieves. Compounds **7**, **21**, and **26** were from Lumiprobe. Reactions were monitored by thin-layer chromatography (TLC) carried out on 0.20 mm Merck silica gel plates (60 F_254_) using UV light as a visualizing agent and an alkaline aqueous solution of KMnO_4_ and heat as developing agents. Merck Silica gel 60 was used for flash column chromatography. Size exclusion chromatography was performed using Sephadex G10 and G50 (Pharmacia Fine Chemicals). UV–Vis absorbance spectra were recorded on a Cary 100 UV-Visible spectrophotometer. Fluorescence spectra were recorded on a Per-kinElmer LS55 luminescence spectrometer. NMR spectra were recorded on Bruker 700 and 800 MHz instruments and calibrated using residual solvent as an internal reference (for CDCl_3_: ^1^H, δ 7.26 ppm and ^13^C, δ 77.16 ppm; for DMSO-*d*_6_: ^1^H, δ 2.50 ppm and ^13^C, δ 39.52 ppm) unless otherwise noted. The following abbreviations were used to explain the multiplicities: s = singlet, d = doublet, t = triplet, q = quartet, m = multiplet, app = apparent, and br = broad. High-resolution mass spectra (HRMS) were recorded on a Thermo Scientific Orbitrap Exactive mass spectrometer using ESI (Electrospray ionization).

### 3.2. Synthetic Procedures

#### 3.2.1. Mesylate **2**

A solution of alcohol **1** [14] (190 mg, 0.43 mmol, 1.0 equiv) in DCM (4.0 mL) was cooled to 0 °C; Et_3_N (87 mg, 0.117 mL, 0.86 mmol, 2.0 equiv) was added, and then MsCl (74 mg, 0.050 mL, 0.64 mmol, 1.5 equiv) was added dropwise. The reaction mixture was allowed to warm to 23 °C and stirred for 1 h. The solvent was then removed under reduced pressure, and the residue was treated with THF (5 mL), upon which, a colorless precipitate formed. The precipitate was separated by filtration, and the THF solution was concentrated under reduced pressure. The resultant residue was purified by flash column chromatography (silica gel, hexane-EtOAc, 10:1→5:1→2.5:1) to yield mesylate **2** as a co-lorless oil (202 mg, 90%). *R*_f_ 0.40 (silica gel, hexane–EtOAc, 1:1). ^1^H NMR (700 MHz, CDCl_3_) δ 4.32 (t, *J* = 6.3 Hz, 2H), 3.50 (t, *J* = 5.8 Hz, 2H), 3.46 (t, *J* = 5.9 Hz, 6H), 3.39–3.34 (m, 14H), 3.01 (s, 3H), 1.99 (p, *J* = 6.1 Hz, 2H), 1.82 (p, *J* = 6.3 Hz, 6H). ^13^C NMR (176 MHz, CDCl_3_) δ 69.9, 69.8, 68.1, 67.3, 66.7, 48.6, 45.5, 37.4, 29.6, 29.2. HRMS (ESI) calcd for C_18_H_36_N_9_O_7_S^+^ [M + H]^+^ 522.2453; found 522.2452.

#### 3.2.2. Imidate **3**

Mesylate **2** (90 mg, 0.17 mmol, 1.0 equiv) was dissolved in a mixture of *t*-BuOH and *i*-PrOH (1 mL, 1:1 *v*/*v*). Ethyl N-hydroxyacetimidate sodium salt (79 mg, 0.63 mmol, 3.7 equiv) was added, and the reaction mixture was refluxed for 2.5 h. It was then cooled down to 23 °C, diluted with EtOAc (10 mL), and filtered through a glass frit. The solvent was removed under reduced pressure, and the resultant residue was purified by flash column chromatography (silica gel, hexane-EtOAc, 15:1→10:1) to yield imidate **3** as a colorless oil (60 mg, 66%). *R*_f_ 0.52 (silica gel, hexane-EtOAc, 2:1). ^1^H NMR (700 MHz, CDCl_3_) δ 4.01 (q, *J* = 7.1 Hz, 2H), 3.95 (t, *J* = 6.4 Hz, 2H), 3.46 (t, *J* = 5.9 Hz, 8H), 3.40–3.33 (m, 14H), 1.92 (s, 3H), 1.87 (p, *J* = 6.4 Hz, 2H), 1.85–1.80 (m, 6H), 1.27 (t, *J* = 7.1 Hz, 3H). ^13^C NMR (176 MHz, CDCl_3_) δ 162.3, 70.7, 69.9, 69.8, 68.5, 68.1, 62.2, 48.7, 45.6, 29.4, 29.2, 14.5, 13.7. HRMS (ESI) calcd for C_21_H_41_N_10_O_6_^+^ [M + H]^+^ 529.3205; found 529.3204.

#### 3.2.3. Phthalimide **5**

Mesylate **2** (101 mg, 0.20 mmol, 1.0 equiv) was dissolved in DMF (2 mL). Potassium phthalimide (51 mg, 0.28 mmol, 1.4 equiv) was added, and the reaction mixture was heated at 90 °C for 2 h. The mixture was then cooled to 23 °C, DMF was removed under reduced pressure (bath temperature: 60 °C), and the resultant residue was suspended in EtOAc (10 mL) and filtered through a pad of Celite (washing with EtOAc). The solvent (EtOAc along with residual DMF) was removed under reduced pressure; the resultant residue was purified by flash column chromatography (silica gel, hexane–EtOAc, 6:1) to yield phthalimide **5** as a colorless oil (102 mg, 92%). *R*_f_ 0.34 (silica gel, hexane–EtOAc, 2:1). ^1^H NMR (700 MHz, CDCl_3_) δ 7.86–7.83 (m, 2H), 7.73–7.69 (m, 2H), 3.77 (app t, *J* = 7.1 Hz, 2H), 3.47–3.42 (m, 8H), 3.39–3.32 (m, 4H), 1.94 (app p, *J* = 6.6 Hz, 2H), 1.82 (app p, *J* = 6.4 Hz, 6H). ^13^C NMR (176 MHz, CDCl_3_) δ 168.5, 134.0, 132.40, 123.3, 70.0, 69.9, 69.0, 68.0, 48.7, 45.5, 35.8, 29.2, 28.9. HRMS (ESI) calcd for C_25_H_37_N_10_O_6_^+^ [M + H]^+^ 573.2892; found 573.2890.

#### 3.2.4. Amine **6**

Phthalimide **5** (78 mg, 0.136 mmol, 1.0 equiv) was dissolved in EtOH (5 mL). N_2_H_4_∙H_2_O (136 mg, 0.133 mL, 2.72 mmol, 20 equiv) was added, and the reaction mixture was stirred at 60 °C for 1 h. The mixture was then cooled to 23 °C, the solvent was removed under reduced pressure, and the resultant residue was suspended in Et_2_O (10 mL) and filtered through a pad of Celite (washing with Et_2_O). The solvent was removed under reduced pressure; the resultant residue was purified by flash column chromatography (silica gel, DCM–MeOH–Et_3_N, 20:1:0→10:1:0→20:1:0.2) to yield amine **6** as a colorless oil (32 mg, 53%). *R*_f_ 0.17 (silica gel, DCM–MeOH–Et_3_N, 5:1:0.06). ^1^H NMR (700 MHz, CDCl_3_) δ 3.49–3.45 (m, 8H), 3.38–3.35 (m, 14H), 2.81 (t, *J* = 6.6 Hz, 2H), 1.82 (m, *J* = 6.3 Hz, 6H), 1.71 (p, *J* = 6.3 Hz, 2H). ^13^C NMR (176 MHz, CDCl_3_) δ 70.1, 70.0, 69.9, 68.1, 48.7, 45.5, 40.0, 33.1, 29.2. HRMS (ESI) calcd for C_17_H_35_N_10_O_4_^+^ [M + H]^+^ 443.2837; found 443.2836.

#### 3.2.5. Imidate **9**

A solution of TSTU (106 mg, 0.353 mmol, 1.2 equiv) in DMF–1,4-dioxane–H_2_O (2:2:1 *v*/*v*/*v*, 5 mL) was added dropwise over the course of 1.5 h to a solution of salt **7** (240 mg, 0.289 mmol, 1.0 equiv) and DIPEA (76 mg, 0.103 mL, 0.588 mmol, 2.0 equiv) in DMF–1,4-dioxane–H_2_O (2:2:1 *v*/*v*/*v*, 12.5 mL). The reaction was stirred at 23 °C for 100 min, and then a solution of oxyamine **8** [15] (122 mg, 0.440 mmol, 1.5 equiv) in DMF–1,4-dioxane–H_2_O (2:2:1 *v*/*v*/*v*, 10 mL) was added. The reaction mixture was stirred for 20 h. After which, it was concentrated to 11 mL and added dropwise to stirred EtOAc (110 mL), upon which, an amorphous purple precipitate was formed. The resultant mixture was passed through a pad of Celitel the precipitate was washed with EtOAc, dried, eluted with MeOH (150 mL), and concentrated. The resultant residue was purified by flash column chromatography (silica gel, DCM–MeOH–H_2_O–Et_3_N, 283:17.5:2:3→184:17.5:2:2→85:17.5:2:1), and then dissolved in H_2_O and purified on a Sephadex G10 column. The resultant solution was passed through a Dowex 50WX4 column in Et_3_NH^+^ form and concentrated. Imidate **9** was obtained as a dark purple amorphous solid (180 mg, 53%). *R*_f_ 0.16 (silica gel, DCM–MeOH–H_2_O–Et_3_N, 85:17.5:2:1). ^1^H NMR (700 MHz, DMSO-*d*_6_) δ 8.36 (t, *J* = 13.4 Hz, 1H), 7.83–7.79 (m, 3H), 7.68 (dt, *J* = 8.2, 1.4 Hz, 2H), 7.40 (dd, *J* = 8.3, 4.7 Hz, 2H), 6.53 (dd, *J* = 13.4, 7.9 Hz, 2H), 4.12 (q, *J* = 8.3 Hz, 4H), 3.93 (q, *J* = 7.0 Hz, 2H), 3.91 (app t, *J* = 4.9 Hz, 2H), 3.57 (app t, *J* = 4.9 Hz, 2H), 3.51–3.45 (m, 8H), 3.37 (t, *J* = 6.0 Hz, 2H), 3.17 (q, *J* = 5.9 Hz, 2H), 3.03 (q, *J* = 7.3 Hz, 9H), 2.21 (t, *J* = 7.2 Hz, 2H), 2.07 (t, *J* = 7.4 Hz, 2H), 1.85 (s, 3H), 1.77–1.66 (m, 16H), 1.59–1.52 (m, 4H), 1.44–1.34 (m, 4H), 1.19 (t, *J* = 7.0 Hz, 3H), 1.16 (t, *J* = 7.3 Hz, 14H). ^13^C NMR (201 MHz, DMSO-*d*_6_) δ 174.3, 174.2, 172.0, 161.7, 149.9, 145.79, 145.77, 141.84, 141.80, 140.08, 140.06, 126.2, 119.8, 110.7, 103.0, 72.3, 69.8, 69.74, 69.70, 69.5, 69.1, 68.4, 61.8, 48.9, 45.7, 43.8, 38.4, 35.0, 33.5, 27.40, 27.38, 26.8, 26.7, 25.7, 25.6, 24.9, 24.2, 14.2, 13.4, 8.8. HRMS (ESI) calcd for C_47_H_66_N_4_O_14_S_2_^2−^ [M–2Et_3_NH]^2−^ 487.2014; found 487.2022.

#### 3.2.6. Triazide **10**

TSTU (8.3 mg, 0.027 mmol, 1.2 equiv) was added to a solution of **9** (27 mg, 0.023 mmol, 1.0 equiv) and DIPEA (7.2 mg, 9.7 µL, 0.056 mmol, 2.5 equiv) in DMF (0.36 mL). The reaction mixture was stirred for 50 min, and then a solution of amine 6 (12 mg, 0.027 mmol, 1.2 equiv) in DMF (0.20 mL) was added. The reaction was stirred for 17 h, and then the mixture was added dropwise to stirred Et_2_O (10 mL), upon which, an amorphous magenta precipitate was formed. The resultant mixture was passed through a pad of Celite; the precipitate was washed with Et_2_O, dried, eluted with MeOH (20 mL), and concentrated. The resultant residue was purified by flash column chromatography (silica gel, DCM–MeOH–H_2_O–Et_3_N, 283:17.5:2:3→184:17.5:2:2), and then dissolved in H_2_O and purified on a Sephadex G10 column. The resultant solution was passed through a Dowex 50WX4 column in Et_3_NH^+^ form and concentrated. The desired compound **10** was obtained as a dark magenta amorphous solid (28 mg, 81%). *R*_f_ 0.21 (silica gel, DCM–MeOH–H_2_O–Et_3_N, 85:17.5:2:1). ^1^H NMR (700 MHz, CDCl_3_) δ 10.65 (br s, 1H), 8.38 (t, *J* = 13.2 Hz, 1H), 7.98–7.88 (m, 4H), 7.11 (dd, *J* = 8.3, 6.6 Hz, 2H), 6.82 (dt, *J* = 27.1, 5.6 Hz, 2H), 6.47 (d, *J* = 13.4 Hz, 2H), 4.12–4.04 (m, 4H), 4.00 (app t, *J* = 4.9 Hz, 2H), 3.96 (q, *J* = 7.1 Hz, 2H), 3.67 (app t, *J* = 4.9 Hz, 2H), 3.64–3.57 (m, 8H), 3.53 (t, *J* = 5.6 Hz, 2H), 3.45–3.41 (m, 6H), 3.41–3.37 (m, 4H), 3.35–3.31 (m, 14H), 3.26 (q, *J* = 6.6 Hz, 2H), 3.17 (qd, *J* = 7.2, 3.1 Hz, 9H), 2.21 (t, *J* = 7.4 Hz, 4H), 1.90 (s, 3H), 1.85–1.76 (m, 10H), 1.74 (p, *J* = 6.8 Hz, 2H), 1.71–1.65 (m, 16H), 1.53–1.44 (m, 4H), 1.38 (t, *J* = 7.3 Hz, 14H), 1.23 (t, *J* = 7.1 Hz, 3H). ^13^C NMR (201 MHz, CDCl_3_) δ 174.8, 173.1, 173.0, 162.8, 150.9, 144.40, 144.37, 142.6, 140.3, 127.6, 120.7, 110.7, 103.8, 72.9, 70.7, 70.63, 70.61, 70.3, 70.0, 69.9, 69.82, 69.80, 69.5, 68.0, 62.3, 49.52, 49.50, 48.6, 46.4, 45.5, 44.73, 44.66, 39.2, 37.5, 36.2, 36.0, 29.6, 29.1, 28.2, 27.3, 27.2, 26.5, 26.4, 25.4, 25.2, 14.5, 13.9, 9.0. HRMS (ESI) calcd for C_64_H_99_N_14_O_17_S_2_^−^ [M–Et_3_NH]^−^ 1399.6760; found 1399.6739.

#### 3.2.7. Monoazide **14**

A solution of TSTU (106 mg, 0.353 mmol, 1.2 equiv) in a DMF–1,4-dioxane–H_2_O mixture (2:2:1 *v*/*v*/*v*; 6.6 mL) was added dropwise to a solution of salt **15** (244 mg, 0.294 mmol, 1.0 equiv) and DIPEA (76 mg, 0.102 mL, 0.588 mmol, 2.0 equiv) in DMF–1,4-dioxane–H_2_O (2:2:1 *v*/*v*/*v*; 6.6 mL). The reaction was stirred at 23 °C for 3 h, and then a solution of aminoazide **16** [26] (96 mg, 0.440 mmol, 1.5 equiv) in DMF–1,4-dioxane–H_2_O (2:2:1 *v*/*v*/*v*; 6.6 mL) was added dropwise. The reaction mixture was stirred for 20 h, after which, it was concentrated under reduced pressure until its volume was brought down to 8 mL. The mixture was then added dropwise to stirred EtOAc (80 mL), upon which, an amorphous magenta precipitate was formed. The resultant mixture was passed through a pad of Celite; the precipitate was washed with EtOAc, dried, eluted with MeOH (150 mL), and concentrated. The resultant residue was purified by flash column chromatography (silica gel, DCM–MeOH–H_2_O–Et_3_N, 283:17.5:2:3→184:17.5:2:2→85:17.5:2:1) then dissolved in H_2_O and purified on a Sephadex G10 column. The resultant solution was passed through a Dowex 50WX4 column in Et_3_NH+ form and concentrated. Monoazide **14** was obtained as a dark magenta amorphous solid (170 mg, 52%). *R*_f_ 0.25 (silica gel, DCM–MeOH–H_2_O–Et_3_N, 85:15:1:4). ^1^H NMR (700 MHz, DMSO-*d*_6_) δ 8.36 (t, *J* = 13.4 Hz, 1H), 7.80 (m, 3H), 7.68 (dt, *J* = 8.2, 1.6 Hz, 2H), 7.39 (dd, *J* = 8.3, 4.5 Hz, 2H), 6.52 (dd, *J* = 13.4, 7.4 Hz, 2H), 4.12 (q, *J* = 8.1 Hz, 4H), 3.58 (app t, *J* = 5.0 Hz, 2H), 3.55–3.46 (m, 8H), 3.39–3.35 (m, 4H), 3.17 (q, *J* = 5.9 Hz, 2H), 3.07 (q, *J* = 7.3 Hz, 6H), 2.21 (t, *J* = 7.2 Hz, 2H), 2.07 (t, *J* = 7.3 Hz, 2H), 1.78–1.67 (m, 4H), 1.71 (s, 12H), 1.60–1.53 (m, 4H), 1.45–1.33 (m, 4H), 1.17 (t, *J* = 7.3 Hz, 9H). ^13^C NMR (201 MHz, DMSO-*d*_6_) δ 174.3, 174.2, 172.0, 149.9, 145.89, 145.87, 141.79, 141.76, 140.06, 140.04, 126.2, 119.8, 110.7, 102.9, 69.74, 69.72, 69.6, 69.5, 69.2, 69.1, 50.0, 48.9, 45.8, 43.8, 38.4, 35.0, 33.4, 27.39, 27.37, 26.8, 26.7, 25.7, 25.6, 24.9, 24.2, 8.7. HRMS (ESI) calcd for C_43_H_58_N_6_O_12_S_2_^2−^ [M−2Et_3_NH]^2−^ 457.1783; found 457.1785.

#### 3.2.8. Imidate **15**

TSTU (40 mg, 0.13 mmol, 1.4 equiv) was added to a solution of **17** (108 mg, 0.096 mmol, 1.0 equiv) and DIPEA (33 mg, 44 µL, 0.25 mmol, 2.6 equiv) in DMF (2.0 mL). The reaction mixture was stirred for 30 min, and then a solution of aminoimidate **19** [10] (37 mg, 0.13 mmol, 1.4 equiv) in DMF (0.30 mL) was added. The reaction was stirred for 18 h, and then the mixture was added dropwise to stirred EtOAc (25 mL), upon which, an amorphous magenta precipitate was formed. The resultant mixture was passed through a pad of Celite, and the precipitate was washed with EtOAc, dried, eluted with MeOH (50 mL), and concentrated. The resultant residue was purified by flash column chromatography (silica gel, DCM–MeOH–H_2_O–Et_3_N, 283:17.5:2:3→184:17.5:2:2) then dissolved in H_2_O and purified on a Sephadex G10 column. The resultant solution was passed through a Dowex 50WX4 column in Et_3_NH^+^ form and concentrated. The desired compound **15** was obtained as a dark magenta amorphous solid (46 mg, 37%). *R*_f_ 0.51 (silica gel, DCM–MeOH–H_2_O–Et_3_N, 85:15:1:4. ^1^H NMR (700 MHz, CDCl_3_) δ 9.96 (br s, 1H), 8.38 (t, *J* = 13.3 Hz, 1H), 7.98–7.88 (m, 4H), 7.11 (dd, *J* = 8.4, 3.8 Hz, 2H), 6.76 (q, *J* = 6.1 Hz, 2H), 6.46 (d, *J* = 13.3 Hz, 2H), 4.16–4.03 (m, 4H), 4.00 (t, *J* = 4.9 Hz, 2H), 3.96 (q, *J* = 7.1 Hz, 2H), 3.66 (t, *J* = 4.9 Hz, 2H), 3.65–3.55 (m, 18H), 3.52 (t, *J* = 5.6 Hz, 4H), 3.38 (q, *J* = 5.6 Hz, 4H), 3.35 (t, *J* = 5.1 Hz, 2H), 3.25–3.15 (m, 6H), 2.20 (t, *J* = 7.3 Hz, 4H), 1.89 (s, 3H), 1.86–1.75 (m, 4H), 1.75–1.59 (m, 4 H), 1.69 (s, 12H), 1.51–1.43 (m, 4H), 1.37 (t, *J* = 7.3 Hz, 9H), 1.22 (t, *J* = 7.1 Hz, 3H). ^13^C NMR (201 MHz, CDCl_3_) δ 174.9, 174.8, 173.2, 173.1, 162.7, 150.9, 144.43, 144.40, 142.6, 140.3, 127.5, 120.7, 110.7, 110.6, 103.74, 103.71, 72.82, 70.70, 70.66, 70.62, 70.60, 70.59, 70.3, 70.2, 70.1, 69.79, 69.77, 69.5, 62.3, 50.8, 49.5, 46.6, 44.7, 39.2, 36.02, 36.00, 28.2, 27.3, 26.4, 25.23, 25.21, 14.5, 13.8, 8.9. HRMS (ESI) calcd for C_55_H_83_N_8_O_16_S_2_^−^ [M−Et_3_NH]^−^ 1175.5374; found 1175.5375.

#### 3.2.9. Conjugate **18**

In a 1.5 mL polypropylene microcentrifuge tube with a screw cap, **14** (3.0 mg, 2.9 μmol, 1.0 equiv) and **17** (2.0 mg, 2.8 μmol, 1.0 equiv) were dissolved in dry DMSO (300 μL). Ascorbic acid (284 mM in H_2_O, 49 μL, 13.9 μmol, 5.0 equiv) and a solution containing CuSO_4_ and TBTA (DMSO–H_2_O 11:10, 10 mM CuSO_4_, 11mM TBTA, 279 μL, 2.8 μmol, 1.0 equiv CuSO_4_) were added, and the reaction mixture was agitated on an orbital shaker at 23 °C for 3 h. The mixture was then poured into 8.4 mL of EtOAc, which resulted in a biphasic mixture. The layers were separated by centrifugation and decantation. The aqueous layer was washed with another 11.5 mL of EtOAc, yielding a dark purple precipitate. The precipitate was washed with 11.5 mL EtOAc using sonication, separated by centrifugation, dried in vacuo, and purified by flash column chromatography (silica gel, DCM–MeOH–H_2_O–Et_3_N, 185:17.5:2:2→270:35:4:3→355:52.5:6:4) to afford conjugate **18** (3.0 mg, 59%) as a dark purple solid. *R*_f_ 0.33 (silica gel, DCM–MeOH–H_2_O–Et_3_N, 60:40:2:1). HRMS (ESI) calcd for C_78_H_99_N_9_O_19_S_4_^2−^ [M−3Et_3_NH+H]^2−^ 796.7976; found 796.7979.

#### 3.2.10. General Procedure for Oxyamine Deprotection

In a 2 mL polypropylene microcentrifuge tube, a solution of HCl in MeOH–H_2_O (0.10 mL, 1.2 M, 12:1 *v*/*v*) was added to a solution of 5 mg of a protected oxyamine in MeOH (1.0 mL). The mixture was vortexed and allowed to stand for 5 min at 23 °C. As TLC analysis indicated complete conversion, the reaction mixture was transferred to a flask and concentrated under reduced pressure. The residue was coevaporated with MeOH (2 × 2 mL) and DCM (2 mL), and then dried under high vacuum. The resultant oxyamine salts were used without further purification.

### 3.3. Synthesis of Antibody Conjugates

#### 3.3.1. General Procedure A

Aqueous NaIO_4_ (11.8 μL, 360 mM) was added to 200 μL of a solution of 6H8 in 20 mM acetate buffer (pH 5.0, 150 mM NaCl). The mixture was agitated on an orbital shaker for 30 min at 23 °C in the dark, and then aqueous glycerol (11.8 μL, 20% *w*/*v*) was added, and the oxidized antibody was immediately purified on a Sephadex G-50 column equilibrated in 20 mM acetate buffer containing Tween 80 (pH 4.0, 150 mM NaCl, 0.01% (*v*/*v*) Tween 80). The concentration of oxidized antibody was measured spectrophotometrically, and immediately after that, the oxidized antibody was added to an oxyamine salt (solution in DMSO). The reaction mixture was agitated on an orbital shaker for 1 h at 23 °C, after which, the conjugate was purified on a Sephadex G-50 column equilibrated in PBST (pH 7.4, 0.01% or 0.05% (*v*/*v*) Tween 80). The yield and stoichiometry of the conjugate were determined spectrophotometrically.

##### Conjugate **20**

According to general procedure A (buffer for purification: 0.01% PBST), 6H8 (0.40 mg, 2.7 nmol, 1.0 equiv) was modified with reagent **12** (20 mM in DMSO, 2.0 μL, 40 nmol, 15 equiv) to yield 0.15 mg (1.0 nmol, 37%) of conjugate **20**.

##### Conjugate **23**

According to modified general procedure A (oxime ligation was performed in acetate buffer containing 0.05% (*v*/*v*) Tween 80, buffer for purification: 0.05% PBST), 6H8 (0.18 mg, 1.2 nmol, 1.0 equiv) was modified with reagent **4** (20 mM in DMSO, 1.0 μL, 20 nmol, 17 equiv) to yield 0.062 mg (0.41 nmol, 34%) of conjugate **23**.

##### Conjugate **25**

According to general procedure A (buffer for purification: 0.01% PBST), 6H8 (0.40 mg, 2.7 nmol, 1.0 equiv) was modified with reagent **16** (5.0 mM in DMSO, 4.0 μL, 20 nmol, 7.4 equiv) to yield 0.11 mg (0.73 nmol, 28%) of conjugate **25** with an average degree of modification of 3.7.

##### Conjugate **28**

According to modified general procedure A (oxime ligation was performed in acetate buffer containing 0.05% (*v*/*v*) Tween 80, buffer for purification: 0.05% PBST), 6H8 (0.18 mg, 1.2 nmol, 1.0 equiv) was modified with reagent **11** (6.1 mM in DMSO, 1.1 μL, 6.7 nmol, 5.6 equiv) to yield 0.11 mg (0.73 nmol, 61%) of conjugate **28** with an average degree of modification of 4.2.

#### 3.3.2. General Procedure B

A solution of a DBCO dye derivative in DMSO was added to a solution of a 6H8 conjugate in 200 μL, PBST (pH 7.4, 0.01% or 0.05% (*v*/*v*) Tween 80). The mixture was agitated on an orbital shaker for 2 h at 23 °C, after which the resultant conjugate was purified on a Sephadex G-50 column equilibrated in PBST (pH 7.4, 0.01% or 0.05% (*v*/*v*) Tween 80). The yield and degree of labeling of the conjugate were determined spectrophotometrically.

##### Conjugate **22**

Conjugate **20** (0.15 mg, 1.0 nmol, 1.0 equiv) was treated with reagent **21** (6.2 mM in DMSO, 16.5 μL, 102 nmol, 102 equiv) according to general procedure B (buffer for purification: 0.01% PBST) to yield 0.075 mg (0.49 nmol, 49%) of conjugate **22** with an average degree of labeling of 3.7.

##### Conjugate **24**

Conjugate **23** (0.053 mg, 0.35 nmol, 1.0 equiv) was treated with reagent **21** (11.1 mM in DMSO, 14.5 μL, 161 nmol, 460 equiv) according to general procedure B (buffer for purification: 0.05% PBST). Due to the high degree of labeling of conjugate **24**, it is impossible to determine its precise quantity and degree of labeling. In assumption that the reaction was quantitative, it yielded 0.053 mg (100%) of **24** with an average degree of labeling of 21.

##### Conjugate **27**

Conjugate **25** (0.11 mg, 0.73 nmol, 1.0 equiv) was treated with reagent **26** (1.78 mM in DMSO, 7.0 μL, 12.5 nmol, 17 equiv) according to general procedure B (buffer for purification: 0.01% PBST) to yield 0.081 mg (0.54 nmol, 74%) of conjugate **27** with an average degree of labeling of 3.0 as measured by sCy5.

##### Conjugate **29**

Conjugate **28** (0.044 mg, 0.29 nmol, 1.0 equiv) was treated with reagent **26** (17 mM in DMSO, 11 μL, 187 nmol, 645 equiv) according to general procedure B (buffer for purification: 0.05% PBST) to yield 0.031 mg (0.21 nmol, 70%) of conjugate **29** with an average degree of labeling of 7.4 as measured by sCy5.

### 3.4. Flow Cytometry

Cell lines THP-1, K562, MelP, and WI-38 with PRAME overexpression were used for staining with labeled antibodies. Before staining, the cells were washed from the culture medium in 1% BSA PBS solution. After washing, the cells were diluted to a concentration of approximately 500,000 per mL. Staining was performed in samples of 100 μL of cell suspension containing 1 μg of antibody conjugate. Incubation was performed for 15 min in the dark. After that, unbound antibodies were washed away with 1% BSA solution in PBS. Flow cytometry was performed on an ACEA NovoCyte instrument; fluorescence was registered in the FITC and APC channels. Table 3 shows the most appropriate channels for each of the dyes.

### 3.5. ELISA Assay

A high protein-binding capacity 96-well ELISA plate was coated with recombinant PRAME protein at a concentration of 100 ng in 100 μL of PBS per well. The plate wells were triple washed with PBST, after which, the 6H8 antibody and its derivatives (100 μL per well in PBS with 1% BSA) were added in duplicate at different concentrations. Following incubation on an orbital shaker for 1 h at 23 °C and three PBST washes, antimouse Fc-specific HRP-labeled antibodies (100 μL per well in PBS with 1% BSA) were added to the wells. After incubation on an orbital shaker for 1 h at 23 °C and triple washing with PBST, a solution of OPD (100 μL per well) was added. The reaction was stopped by adding 10% sulfuric acid, and the optical density (OD) was measured at 490 nm on a Packard SpectraCount BS10000 (PerkinElmer, Waltham, MA, USA) microplate reader.

## 4. Conclusions

We have developed a methodology that allows for producing fluorescent antibodies with a higher degree of labeling. It relies on the periodate oxidation of IgG glycans followed by oxime ligation with branched linkers and fluorescent labeling by SPAAC. Antibody affinity was found to be largely unaffected in the course of the synthesis. The utility of the approach was demonstrated by the detection of the PRAME protein in cell membranes by flow cytometry. The results of this work can be used as the basis for the synthesis of branched antibody-based conjugates, such as fluorescent probes and antibody–drug conjugates with an increased drug load.

## Figures and Tables

**Table 1:** Molar absorbance coefficients of the conjugate components.

	ε_280_, M^−1^cm^−1^	ε_max long wave_, M^−1^cm^−1^
6H8	2.10 × 10^5^	-
FAM-DBCO	2.17 × 10^4^	7.40 × 10^4^
sCy3	9.72 × 10^3^	1.62 × 10^5^
sCy5-DBCO	1.08 × 10^4^	2.71 × 10^5^

**Table 2:** Median fluorescence intensity.

	Unstained, FITC	22	24	Unstained, APC	27	29
K-562	161	219	192	226	1956	3223
THP-1	170	234	203	216	1447	3384
MelP	186	316	267	234	3521	10,477
WI-38 PRAME+	148	676	404	208	27,267	56,987

**Table 3:** Dyes and suitable channels for their detection.

Dye	Extinction	Emission	Laser	Detector
FAM	488	515	488	530/30 FITC
Cy5	643	667	640	675/30 APC

## Data Availability

Not applicable.

## Sample Availability

Samples of the compounds are available from the authors.
